# An overview of mammographic density and its association with breast cancer

**DOI:** 10.1007/s12282-018-0857-5

**Published:** 2018-04-12

**Authors:** Shayan Shaghayeq Nazari, Pinku Mukherjee

**Affiliations:** Department of Biological Sciences, University of North Carolina at Charlotte, University City Blvd 9201, Charlotte, NC 28223-0001 USA

**Keywords:** Breast cancer, Dense breast tissue, Mammographic density, Extracellular matrix stiffness, Mammography

## Abstract

In 2017, breast cancer became the most commonly diagnosed cancer among women in the US. After lung cancer, breast cancer is the leading cause of cancer-related mortality in women. The breast consists of several components, including milk storage glands, milk ducts made of epithelial cells, adipose tissue, and stromal tissue. Mammographic density (MD) is based on the proportion of stromal, epithelial, and adipose tissue. Women with high MD have more stromal and epithelial cells and less fatty adipose tissue, and are more likely to develop breast cancer in their lifetime compared to women with low MD. Because of this correlation, high MD is an independent risk factor for breast cancer. Further, mammographic screening is less effective in detecting suspicious lesions in dense breast tissue, which can lead to late-stage diagnosis. Molecular differences between dense and non-dense breast tissues explain the underlying biological reasons for why women with dense breasts are at a higher risk for developing breast cancer. The goal of this review is to highlight the current molecular understanding of MD, its association with breast cancer risk, the demographics pertaining to MD, and the environmental factors that modulate MD. Finally, we will review the current legislation regarding the disclosure of MD on a traditional screening mammogram and the supplemental screening options available to women with dense breast tissue.

## Introduction

Breast cancer is the leading cause of cancer-related mortality worldwide, with the most incidents occurring in the United States and in Western Europe [1]. Scientists have made great progress in the development of better diagnostic and treatment methods for breast cancer, which have contributed significantly to the drop in the mortality rate. However, this malignancy still accounts for more than 500,000 deaths annually worldwide [1]. A major risk factor contributing to the breast cancer burden is the presence of mammographic dense breast tissue. In fact, more than 50% of women under the age of 50 years have high MD [2].

**Fig. 1:**
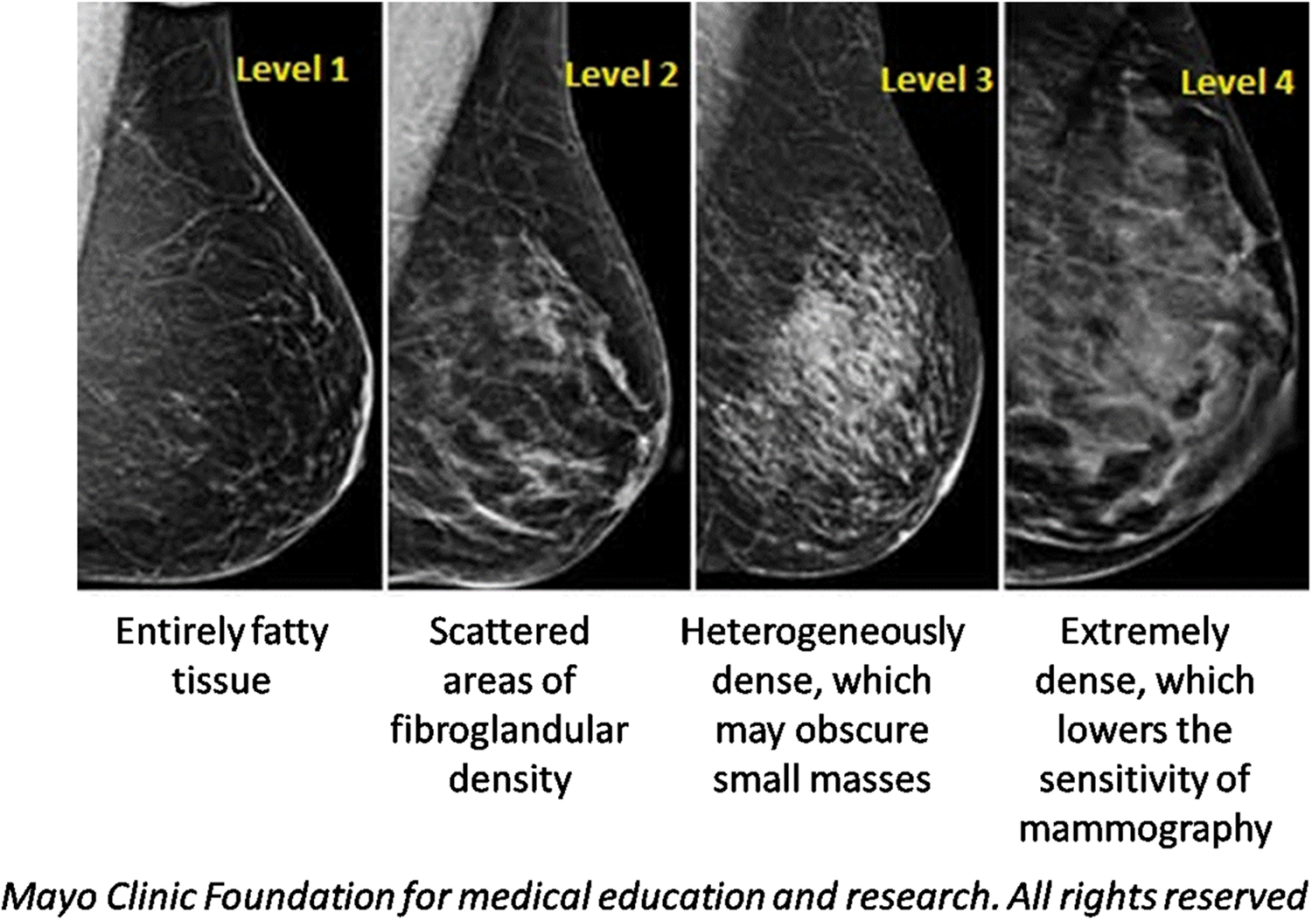
The visual classification associated with mammographic density [74]. Level 1—breast tissue consisting of entirely adipose tissue with almost no dense tissue. Level 2—scattered density with mostly fat tissue. Level 3—heterogeneous distribution of dense tissue with little fat in the breast tissue. Level 4—highly dense tissue with little to no adipose tissue [74]. (This figure is adapted from an article published by Mayo Clinic with their formal permission to be used in this manuscript)

Mammographic density refers to the percentage of dense tissue of an entire breast. The percent mammographic density (PMD) is based on the appearance of MD in accordance to the different X-ray attenuation characteristics of breast tissue composition [3]. Fat is radiologically translucent, so X-rays can pass through it unhindered, making it appear darker on a mammogram. Epithelial and connective tissue, including the glands, are radiologically dense, and block X-rays more than fat tissue, so as a result they appear white on a mammogram [4]. MD is, therefore, defined as fibroglandular mammary tissue consisting of fibroblasts, epithelial cells and connective tissue [5]. The most commonly used tool for assessing MD on a mammogram is the breast imaging reporting and data systems (BI-RADS) [6]. The BI-RADS divides MD into four major categories, as illustrated in Fig. 1 (adapted from an article published by Mayo Clinic with their formal permission to be used in this manuscript). Level one defines an almost entirely fatty breast tissue with 5–24% tissue density (10% of women in the US), while level two defines a breast tissue composed of scattered areas of density at 25–49%, but still composed of mainly fatty tissue (40% of women in US). The third level, described as heterogeneous density, indicates areas of non-dense tissue with 50–75% tissue density (40% of women in US). Finally, level four is composed mostly of ≥ 75% tissue density with very little to no fatty tissue, and is designated as extremely dense (10% of women in US) [7]. Women with heterogeneously or extremely dense breast tissue (50% of women in the US) are considered to have high MD. Other methods used for assessing MD include automatic volumetric measurements using the software package Volpara™ and PMD using Cumulus [8, 9].

## Mammographic density as a double jeopardy

Mammographic density (MD) poses two major problems for women who have it. First, MD decreases the detection sensitivity of screening mammography; second, MD is an independent risk factor for breast cancer. Furthermore, women with highly dense breasts are shown to be at greater risk for developing breast cancer during their lifetime, compared to women with low dense breast tissue.

### Mammographic density masks breast cancer

Mammography still remains the most widely used method for the detection of breast cancer. Yet, recent studies have revealed the limitations of mammography, especially in women with high-density breast tissue. A standard screening mammogram cannot detect all cancers because the sensitivity of mammogram depends on the density of the breast tissue [10]. On a screening mammogram, radiologically dense tissue appears white. The lack of contrast between cancer and the breast background tissue (dense tissue) makes it more difficult to detect breast cancer on a mammogram with dense breasts. This means that women with dense breasts are more likely to experience both false positives and false negatives in mammography interpretations [11]. Kolb et al. conducted a study of 11,130 women who were asymptomatic for breast cancer and underwent mammographic screenings, and found that sensitivity of mammogram declined to 48% in women with extremely dense breasts compared to the entire sample of women in this study, who had a 78% mammographic sensitivity [12]. In another retrospective study analyzing 8 years of screening mammograms from 329 breast cancer patients (335 total breast cancers) with type 2–4 MD category, only 19% of cancers were identified by 3 or more out of the 5 blinded radiologists. 81% of the breast cancers were missed after screening mammography [13]. When the results were re-studied by 3 unblinded radiologists, 78% of the breast cancers were considered to be obscured because of the overlap of the dense tissue [13]. These results support the notion that having highly dense breast tissue can interfere with the early detection goal of screening mammography and thereby make the mammogram results of women with higher density breast tissue inconclusive.

### Mammographic density as an independent risk factor for breast cancer

The presence of dense breast tissue greatly and independently increases the risk for developing breast cancer [14]. Wolfe was the first researcher to observe and publish the association between the presence of dense breast tissue and the occurrence of breast cancer [15, 16]. Since then, several studies have confirmed this positive correlation between MD and the risk of developing breast cancer [17–34]. In a large meta-analysis conducted by McCormack and colleagues [35] which compared percent density and breast cancer incidence, the combined relative risk of breast cancer was 1.79 (1.48–2.16), 2.11 (1.70–2.63), 2.92 (2.49–3.42), and 4.64(3.64–5.91) for MD categories 5–24% (level 1), 25–49% (level 2), 50–74% (level 3), and ≥ 75% (level 4), respectively. These data suggest that there is a strong positive association between the increase in MD and the increased risk for breast cancer. Additionally, Boyd and researchers have speculated that because of this strong association, out of all the breast cancer cases reported, one-third could be linked to the existence of highly dense breast tissue [36]. However, the underlying mechanisms of the positive association between MD and the risk of breast cancer remain to be elucidated.

In this review article, we will summarize a few of the environmental and genetic factors that can influence MD, and ultimately play a role in breast cancer initiation and progression. Additionally, we highlight the problems with detecting breast cancer on a traditional screening mammogram and provide an overview of the alternative screening options, including a novel antibody.

## Factors that can influence mammographic density

### Heritability and mammographic density

Mammographic density is shown to be heritable in a couple of studies [14, 37]. In a study comparing PMD among monozygotic and dizygotic twins, after adjustments for age and additional covariates, the correlation coefficient between PMD was about twice as high in monozygotic (0.63) compared to dizygotic twins (0.27) [14]. Similarly, another study [37] showed higher percent and absolute mammographic density in monozygotic twins (correlation coefficient 0.74) compared to dizygotic twins (correlation coefficient 0.38) [37]. These studies highlight the importance of genetic components in MD. However, it is important to note that it is still unknown whether this heritable effect is influenced by non-heritable environmental factors, as well as factors related to an individual’s behaviors [37].

### Parity status and number of births

In one study, parity status and number of births were significantly and inversely associated with percent collagen in the breast tissue/breast tissue density [38]. Smaller breasts were also reported to be associated with a greater amount of collagen and glandular tissue [38].

### Race and ethnicity

In a large study [39] including Asian, Caucasian, African American, and “other ethnicities,” the greatest MD was seen in Asian women and the lowest MD in African American women [39]. Two additional studies also showed that MD is significantly higher in women of Chinese ethnicity [40] compared to other ethnic groups [41]. While race and ethnicity may be driving factors for breast tissue density, it is not fully understood if the difference in MD in different racial groups explains the differences in breast cancer risks [39]. Clearly, factors such as diet and environmental exposures have significant influence on the risk of developing breast cancer in the various ethnic groups.

### Diet

Mammographic density (MD) can differ in women when comparing dietary differences. In one study [42], women with a higher dependence on western diet patterns had higher MD compared with women with low dependence on this diet [42]. Another study of postmenopausal Japanese women found a significant positive association between PMD and intake of protein and fats after controlling for covariates [43]. In addition to the contribution of diet to MD, alcohol intake can also modulate MD. Women who consume more than 7 alcohol servings per week, especially those with a BMI of less than 25 kg/m^2^, have a 17% higher PMD compared to non-drinkers [44]. Together, these studies suggest that dietary factors could have an implication in the risk of breast cancer by contributing to the increase in MD.

### Hormonal replacement therapies (HRT)

Hormonal replacement therapies and treatment with tamoxifen such as combination of estrogen and progesterone as well as treatments with tamoxifen are known to increase MD [45]. However, estrogen therapy alone does not significantly increase MD [45]. Previous reports [46, 47] have found a positive correlation between MD and HRT, which resembles the well-studied relationship between HRT and breast cancer risk [47]. Treatment with tamoxifen, which blocks estrogen receptors, has been shown to decrease MD in the short term, but not in the long-term [48]. In a study [48] that included 818 healthy women at high risk for breast cancer, after 18 months of treatment with tamoxifen or placebo, there was a 7.3% reduction in MD in the tamoxifen group compared to a 3.5% decrease in MD in the placebo group. Although this trend continued after 54 months of treatment, the group on the tamoxifen regimen had a 28.2% reduction in MD from baseline, and the placebo group’s density was reduced to 35.3% from baseline. This study demonstrated that there was a significant reduction in MD in the tamoxifen group due to the therapy. However, other environmental factors could also play a role in decreasing MD [48]. Interestingly, in a randomized breast cancer prevention trial, women who were on tamoxifen treatment and had a 10% reduction in MD experienced a 63% reduction in breast cancer risk; however, those women who were taking tamoxifen, but experienced less than 10% or no reduction in MD had no decrease in breast cancer risk [49]. We could conclude from these results that an 18-month regimen of tamoxifen may reduce MD as well as reduce the risk for breast cancer.

Taken together, these findings suggest that several factors, including race, genetics, parity, menopausal status, HRT, and diet can modulate MD, and can, therefore, have an effect on a woman’s risk for breast cancer. However, additional studies need to be done to support these findings.

## MD, breast cancer risk, and molecular subtypes of breast cancer

Data from 6 studies showed a positive association between MD and the risk for invasive tumors across all ages, where the highest level of dense tissue showed a twofold increase in risk compared to the average level of dense tissue [50]. In one study, there was a positive association between MD and ER^−^ HER2^−^ breast cancers in women younger than 55 compared to women who were older than 55 years of age [50]. Human epidermal growth factor receptor 2 (HER2) is a member of the epidermal growth factor receptor (EGFR) family and is overexpressed in about 30% of invasive breast cancers. High MD is strongly associated with large tumors, positive lymph nodes, and ER^−^ tumors in women younger than 55 years of age [50]. This suggests that MD could potentially play a role in the aggressiveness of breast cancers; however, more controlled studies need to be conducted to confirm these observations [50]. In another study that included 733 women with invasive breast cancers [51], there was a higher association of MD with ER-negative tumors, including triple-negative breast cancer (TNBC) cases, compared to luminal A breast cancers [51]. An association between high MD and androgen receptor (AR)-negative tumors was also observed, but this association was reported to be weak [51]. Future studies need to address and confirm MD and its association with subtypes and aggressiveness of the breast cancer.

## Extracellular matrix (ECM) proteins affecting breast tissue density

The tumor microenvironment, which is integrated within the ECM, consists of several cell types, such as endothelial cells, smooth muscle cells, carcinoma-associated fibroblasts (CAFs), and immune cells. The ECM, sometimes referred to as stroma, is a complex and dynamic matrix that includes proteins such as laminin, fibronectin, collagen, proteoglycans (PGs), and proteases [52]. These proteins serve as a structural scaffold providing support for tissue assembly, maintenance, and integrity [53]. Recent studies have shown that both stromal architecture and composition can exert an important influence on normal epithelial biology. Further, stromal alterations might not always be ‘reactive’ to epithelial tumor development, but might sometimes play an initial ‘landscaping’ role in breast carcinogenesis.

*Collagen type I* is one of the major components of the stromal ECM network that influences tissue density [54]. Collagen re-organization and crosslinking act as a scaffold aiding cancer cells to migrate and invade surrounding tissue and is thus associated with metastasis and poor prognosis in breast cancer patients [55]. In the presence of three-dimensional collagen, untransformed mammary epithelial cells express high levels of proteins such as MT1-MMP, as well as mesenchymal markers (vimentin, and fibronectin), which are indicative of a malignant phenotype [56].

*Small leucine*-*rich proteoglycans* (SLRPs) also make up a large portion of the ECM, and high levels of PGs increase tissue density and carcinogenesis [52]. Lumican, decorin, fibromodulin, and biglycan are part of the family of SLRPs that have been implicated in increasing tissue density. Lumican is an important protein that plays a role in tissue repair and embryonic development. There is an increased expression of lumican in high density compared to low density tissue [57]. High expression of lumican can induce initiation and progression of breast cancer by increasing angiogenesis, cell growth, migration, and invasion [58]. Higher levels of lumican are associated with higher tumor grade and lower expression of ER receptors in cancer cells [59]. Decorin follows the same expression pattern as lumican, with higher expression in high density versus low density tissue [57]. The roles that high expression of lumican and decoran play in high-density breast tissue are unclear and need further exploration. Currently, decorin is being explored as a chemoprevention drug. With robust studies, lumican could be an attractive target for modulating MD. Better understanding of the molecular interplay between the SLRPs and major oncogenic signaling pathways in dense versus non-dense tissue may lead to the ability to alter tissue density effectively and reduce breast cancer incidence.

## Mammographic density and other oncogenic signaling

Expression of Ki-67, a cell proliferation marker, in high versus low density tissue remains controversial, with few studies suggesting no association, while one study suggested higher Ki-67 in stroma of high versus low density tissue [51, 60, 61]. The authors that found a correlation of tissue density with Ki-67 also reported a decrease in CD44, a TGF-β target and an increase in cyclooxygenase-2 (COX-2) in the stroma of high versus low density breast tissue [60]. These authors concluded that TGF-β repression elevated the expression of COX-2 and Ki-67 in women with high versus low-density breast tissue [60], providing some evidence of why women with high-density breast tissue are at risk of developing breast cancer. Of note is that COX-2 over-expression is clearly associated with invasive breast cancers and ductal carcinoma in situ, but its association with dense tissue has not been fully investigated [62].

## Cross-talk between fibroblasts and epithelial cells in dense tissue

Since highly dense stromal tissue can trigger proliferation in the breast epithelium in women with high MD, there must be cross-talk between stromal cells (fibroblasts) and epithelial cells in a dense microenvironment [63]. Indeed, high density associated fibroblasts (HDAFs) express significantly decreased levels of CD36 compared to Low Density Associated Fibroblasts (LDAFs) in the breast tissue of disease-free women [64]. CD36 is a transmembrane receptor that is involved in adipocyte differentiation, angiogenesis, apoptosis, TGF-β activation, cell-ECM interactions and immune signaling [64]. This decrease in CD36 is particularly significant, because a similar downregulation of CD36 gene expression is observed in carcinoma-associated fibroblasts (CAFs) compared to fibroblasts from reduction mammoplasty (RMF) [64]. These results suggest that the downregulation of CD36 observed in both HDAFs of disease-free women and CAFs, can be an early event in tumor formation [64]. Dense breast tissue also has a greater expression of DNA damage response (DDR) genes and shorter telomere length compared to low-density breast tissue [65]. DDR is associated with an increase in Activin-A expression and a reduction in the expression of PPARγ, a transcription factor regulating CD36 [65]. These genetic and functional differences between the HDAFs and LDAFs are one of the reasons for decreased differentiation of adipocytes in high-density breast tissue.

## The challenge associated with screening highly dense breasts with screening mammograms and supplemental screening options

Screening for breast cancer is predominantly done by mammography and clinical breast exams which has increased the chances of survival. While mammograms have resulted in early diagnosis for many women, 27% of breast cancers are missed in women with dense breasts due to lesion obscuration [66]. Given these challenges, multi-modal screenings offer the best chance of enhancing breast cancer screening effectiveness. Magnetic resonance imaging (MRI), ultrasonography, and digital breast tomosynthesis (DBT) can all be great supplemental tools for breast cancer screening in women with dense breasts, but they all have several disadvantages too. Compared to mammography, ultrasound has high sensitivity to detect breast cancer regardless of breast tissue density; however, the specificity is low, which results in high false-positive rates [67]. However, combining breast cancer screening methods have displayed promising results. In a recent study [66], ultrasonography in adjunction to mammography significantly increased the number of breast cancers detected in women with MD, compared to mammography alone. The combined screening methods detected 27% additional cancers, but the lack of specificity still remains a limitation of this adjunctive therapy [66]. Screening mammography is limited because of its two-dimensional nature. Recent breast cancer screening method includes the DBT which is a three-dimensional (3D) X-ray imaging technology [68] that creates a 3D cross section of the breast tissue, allowing for better all-over visualization of the breast. Therefore, DBT limits the possibility for missing tumors because of the overlap of breast tissue seen in the 2-D imaging of the traditional screening mammogram. It has been observed that women undergoing DBT in addition to mammography had significantly lower false positive cancers reported than women going through digital mammography alone [69]. Unfortunately, DBT uses twice as much radiation as conventional mammography, and most insurance companies are unwilling to pay for the extra cost. Thus, adoption is limited. Another disadvantage is that interpretation of the DBT X-ray images is greatly dependent on the radiologist’s expertise, and is, therefore, highly variable. Thus, there remains a pressing need for the development of additional non-invasive tests that can be used in conjunction with mammography.

## New, emerging biomarker for early detection of breast cancer in women with dense breasts

TAB 004 is an antibody developed to target tumor-associated MUC1 (tMUC1), an antigen that is present at high levels in the serum of cancer patients, including pancreatic and breast cancer [70]. MUC1 is present on the surface of normal cells, and contains extensive *O*-glycan branching on its N-terminus domain. However, in a tumor microenvironment, MUC1 loses its O-glycan branching and dissociates from its C-terminus domain, which is attached by hydrogen bonding. The low glycosylation on tMUC1 exposes its variable number tandem repeat (VNTR) region, which allows TAB 004 to bind and be detected [71]. TAB 004 specifically recognizes tMUC1 across all breast cancer subtypes and is not affected by tissue density. Thus, tMUC1 can serve as a biomarker that can aid in BC diagnosis in women with dense breast tissue. Preclinical and clinical studies have systematically examined the presence of tMUC1 on ~ 450 human breast cancer tissues across all subtypes. In addition, a TAB 004-based ELISA has been developed to monitor circulating levels of tMUC1 in patients with and without breast cancer across high and low density tissue to aid in the early detection of BC in conjunction with mammography [72]. In a longitudinal screening study, the results showed that the tMUC1 biomarker test could detect breast cancer 2 years prior to diagnosis by screening mammography. These clinical studies have led to a CLIA registered Laboratory Developed Test with a commercial name: Agkura™ Personal Score (licensed and marketed by OncoTAb Inc.).

## The controversy surrounding mammographic density reporting and legislative changes

It has been known for decades that MD masks breast cancer on a standard screening mammogram. Yet, only recently the general public and the medical community have started discussing this topic. This is in part due to the great efforts of Dr. Nancy Cappello. Dr. Nancy Cappello was diagnosed with stage III breast cancer after many years of negative mammograms, which failed to report the status of her MD. Because of Dr. Cappello’s great efforts, in 2009 Connecticut became the first state to mandate that mammogram reports must include information regarding the status of a woman’s MD. Dr. Cappello has since started an organization entitled “AreYouDense.org”, (https://www.areyoudense.org/) which has raised awareness about the decrease in mammography sensitivity because of the presence of high MD. The mammography quality standard act (MQSA) which is set forth by the US food and drug administration (FDA), ensures that a written mammography report is sent to each patient. However, currently there is no federal law mandating mammography reports to include information regarding the patient’s MD. Due to this shortcoming, law makers have been passing legislation state-by-state to ensure that these mammography reports include density status [73]. As of January 2018, 30 states have MD notification laws in effect. In an interview, Dr. Cappello highlighted the importance of encouraging the U.S. FDA to make an amendment to the MQSA to include a density notification section to ensure that women nationwide will be notified and educated about the status of their MD (Personal Communication with Dr. Nancy Cappello).

## Concluding remarks

**Fig. 2:**
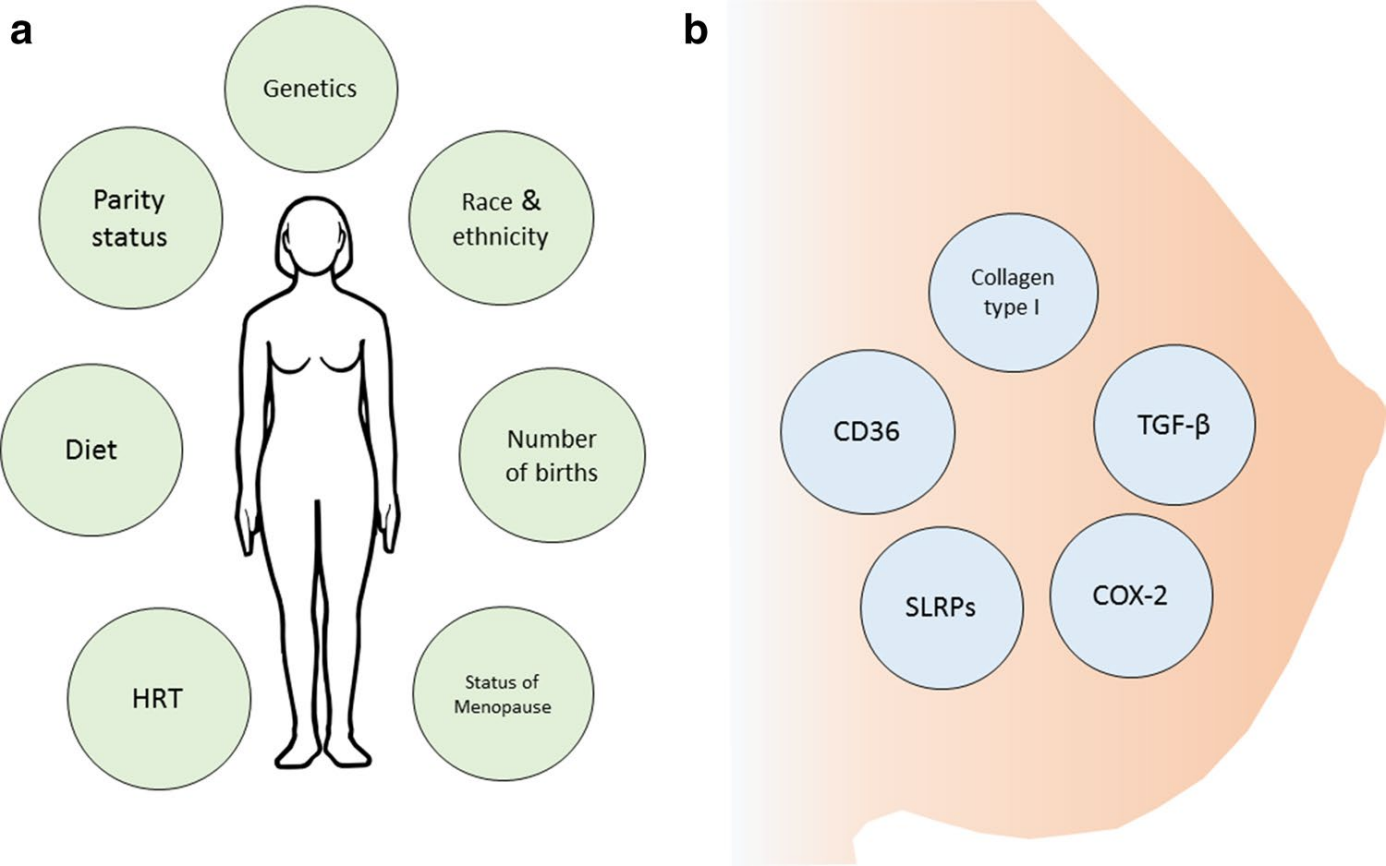
An overview of the factors that can modulate mammographic density. **a** Some of the environmental factors that can affect mammographic density. **b** A few molecules involved in modulating dense tissue in the breast. *HRT* hormonal replacement therapy, *SLRPs* small leucine-rich proteoglycans, *TGF-β* transforming growth factor-β, *COX-2* cyclooxygenase-2, *CD36* cluster of differentiation 36

Women with high-density breast tissue face two major challenges; (a) late diagnosis of breast cancer due to poor sensitivity of mammographic screening and (b) higher risk for developing breast cancer. Although heritable, breast tissue density may be modulated to a certain extent by external factors and therapies, as summarized in Fig. 2. There are many gaps in the understanding of cellular and molecular mechanisms underlying the strong association of dense breast tissue with initiation of breast cancer. There is a critical need to explore the cell-to-cell interactions between epithelial ductal cells and stromal cells in high versus low MD breast tissue. Attention must also be given to the development of robust multimodal screening strategies for women with dense breast to improve the sensitivity of breast cancer detection, including novel imaging modalities along with discovery of circulating biomarkers.
